# Teleconference versus Face-to-Face Scientific Peer Review of Grant Application: Effects on Review Outcomes

**DOI:** 10.1371/journal.pone.0071693

**Published:** 2013-08-07

**Authors:** Stephen A. Gallo, Afton S. Carpenter, Scott R. Glisson

**Affiliations:** American Institute of Biological Sciences (AIBS), Scientific Peer Advisory and Review Services (SPARS) Division, Reston, Virginia, United States of America; Max Planck Society, Germany

## Abstract

Teleconferencing as a setting for scientific peer review is an attractive option for funding agencies, given the substantial environmental and cost savings. Despite this, there is a paucity of published data validating teleconference-based peer review compared to the face-to-face process.

Our aim was to conduct a retrospective analysis of scientific peer review data to investigate whether review setting has an effect on review process and outcome measures.

We analyzed reviewer scoring data from a research program that had recently modified the review setting from face-to-face to a teleconference format with minimal changes to the overall review procedures. This analysis included approximately 1600 applications over a 4-year period: two years of face-to-face panel meetings compared to two years of teleconference meetings. The average overall scientific merit scores, score distribution, standard deviations and reviewer inter-rater reliability statistics were measured, as well as reviewer demographics and length of time discussing applications.

The data indicate that few differences are evident between face-to-face and teleconference settings with regard to average overall scientific merit score, scoring distribution, standard deviation, reviewer demographics or inter-rater reliability. However, some difference was found in the discussion time.

These findings suggest that most review outcome measures are unaffected by review setting, which would support the trend of using teleconference reviews rather than face-to-face meetings. However, further studies are needed to assess any correlations among discussion time, application funding and the productivity of funded research projects.

## Introduction

The American Institute of Biological Sciences (AIBS) has been providing peer review services to the biological research community since 1963 [1], [2]. AIBS has provided these services for a wide variety of clients, including federal and state funding agencies as well as non-governmental organizations and private research foundations [3]. In conducting these grant application reviews, AIBS and others in the review community have noticed a growing trend toward the use of the teleconference and video teleconference review settings as alternatives to face-to-face meetings [4]. Although adoption of teleconferencing and video teleconferencing as settings for peer review has become more attractive to funding agencies, the majority of grant reviews done for the NSF and the NIH still occur via face-to-face panels utilizing tens of thousands of reviewers each year, despite the substantial environmental and cost savings, and the convenience to the reviewers afforded by teleconferences [4]. One potential reason is that there is little published data validating the peer review process in general, and no studies exploring whether the review setting significantly alters the quality of the peer review process [5], [6]. Although recent studies have implied that scientific collaborations are less fruitful (in terms of publication and citation levels) when the collaborators are not co-located, it is unclear whether this has any relevance to the peer review process [7].

Overall, existing data in the psychology and team performance literature seem to indicate that the performance of teams is impacted by technologically mediated communication (teleconferences, chat, email, etc.), however, the extent is dependent on the type of technology, the type of task in which the team is engaged, and whether the team is ad-hoc (temporary) or established (appointed members serving regularly over a prescribed period of time) [8]. In AIBS peer review panels, teams are often ad-hoc (which might make them more susceptible to being influenced by communication technology than established teams) and focus on tasks which are both persuasive (more susceptible) and analytical (less susceptible) [8]. However, two separate studies of team performance (one with ad-hoc teams and one with established teams) in a teleconference setting have shown performance levels to be equal to face-to-face performance (including measures such as time-on-task), in part due to an enhanced task-oriented focus [9], [10]. In terms of conducting peer review of grant applications, to our knowledge there are no results in the literature that relate these findings to the peer review process and no attempts to validate the procedures used for teleconference reviews versus those used for face-to-face reviews.

AIBS has coordinated the scientific peer review of thousands of applications for one specific program (PrX) in support of a federal agency for over a decade, revealing interesting and informative data on the peer review process. Importantly, one aspect of the peer review process for PrX that evolved over the years is the review setting; reviews that were conducted via face-to-face meetings have transitioned to teleconference review meetings. Aside from the change in review setting, most of the AIBS review process for PrX has remained fairly constant. Reviewers have consistently used the same scoring scale, the same rules regarding conflict of interest and the same discussion format. Reviewers have consistently provided specific evaluative information to the client and specific feedback to investigators in much the same format. Therefore, PrX represents an appropriate opportunity for a retrospective study to observe some of the output metrics of the peer review process and examine whether they are affected by the change in review setting from face-to-face to teleconference meetings.

## Background

### Program Funding

Funding for this program is appropriated to support research in a wide variety of topic areas (more than 80 different areas over the last 13 years). Topic areas have included, but are not limited to: vision, drug abuse, nutrition, blood-related cancer, kidney disease, autoimmune diseases, malaria, tuberculosis, osteoporosis, arthritis, and autism research. AIBS has provided independent, objective scientific peer review services for this program since its inception in 1999, reviewing over 6,000 applications. While several funding mechanisms have been employed, the most consistently used mechanism has been an NIH R01-like mechanism, which funds studies over 3-year periods in amounts up to $750,000 in direct costs. AIBS derived the data for this analysis from the review of applications submitted to this mechanism in the years 2009–2012. It should be noted that in both 2010 and 2012, a pre-application cull was used in which only a subset of investigators were invited to submit a full application, thus reducing the number of full applications for those years. The success rate of full applications was 4.6%, 9.3%, 8.9% and 10.1% for 2009–2012, respectively.

### Review Procedures

AIBS staff members recruit subject matter experts to review applications submitted to specific topic areas, choosing reviewers with areas of expertise closely matching the research applications under review. Reviewers are vetted for real and perceived conflicts of interest. They are required to sign a non-disclosure agreement to maintain the confidentiality of the review. Each review panel consists of 7–12 subject matter experts (including a chairperson) and, in recent years, one or more consumer reviewers. Consumer reviewers are full voting members who have direct experience with diseases relevant to the scientific topic areas being evaluated by the peer review panels. All panel members receive online and face-to-face (when applicable) orientations describing the AIBS peer review process.

Once panel members are recruited, review materials are disseminated and panel members begin reading and evaluating applications. Reviewers have access to all the applications but are only responsible for providing written comments for a subset of applications that closely matches their specific subject matter expertise; each application has at least two assigned reviewers. Reviewers score assigned applications using specific review criteria. Each application is given an overall scientific merit score. The overall scientific merit scale is from 1.0 to 5.0 (where 1 is highest scientific merit and 5 is lowest scientific merit). In recent years, reviewers have used the AIBS online evaluation system (SCORES; trademark pending), which allows for the capture of the initial evaluations and scores as well as online conferral among reviewers when needed.

For face-to-face review meetings, participants travel to the meeting destination (usually a hotel) for a one- or two-day meeting, depending on the size and number of applications to be reviewed. No travel is necessary for teleconference reviewers. During the peer review meeting (either face-to-face or teleconference), assigned reviewers present their critiques of the strengths and weaknesses for each application using specific review criteria. The discussion is then open to the panel. AIBS staff and the panel chairperson ensure that each application is reviewed using a consistent process and that overall scientific merit scores reflect what is written in each critique. AIBS staff also ensure that all applications receive a thorough and equitable discussion and that all panel members' concerns are noted. After panel discussion is completed, each panel member submits their final score (using the online AIBS SCORES system) on each application. The electronic score sheets are then locked, time-stamped and the final scores are recorded. The panel then moves on to the next application until all have been discussed and scored. Afterward, an overall summary paragraph of the panel's evaluation of the application's strengths and weaknesses and panel recommendations is created by the assigned reviewers for each application and then approved by the panel chairperson.

Written critiques and summary statements are edited by AIBS staff to ensure scientific accuracy and clarity. The final deliverable to the funding agency for each application consists of the overall summary statement, the average of the panelists' scores (also referred to as the overall score [OS]) and the assigned reviewers' critiques (anonymized). Panel members are then surveyed regarding the quality of review, the review procedures, interactions with AIBS staff, etc., to ensure continuous process improvement for AIBS and its clients.

Until 2011, the peer review for PrX was conducted through face-to-face peer review panels with only occasional teleconference reviewers for applications requiring specialized expertise. In 2011 and 2012, all applications were reviewed via teleconference panels.

### Approach

In this analysis we compare reviewer scoring behavior of 1600 applications over a 4-year period: data from two years of face-to-face panel meetings and two years of teleconference meetings were used. The average overall score (AOS) for applications reviewed each year is recorded. It should be noted that the OS for any given application is an average of individual reviewers' scores, and the AOS is the average of all the OSs of all applications from all panels for that year. The AOS of applications are compared over time, along with average standard deviations and reviewer inter-rater reliability (IRR) measures. The average discussion time per application was recorded per panel and then averaged over all panels for that year. The reviewer demographics were also recorded and analyzed over time. Where applicable, one-way ANOVA was applied with post-hoc Scheffe tests to compare data sets for each year and provide measures of statistical significance. Finally, some results of a reviewer survey to gather reviewers' assessments of the quality of the review process are also provided.

It should be noted that, although the specific scores/details of individual applications must be kept confidential, the data sets collected for this study will be anonymized and made available upon request.

## Results and Discussion

### Application Scoring

**Figure 1:**
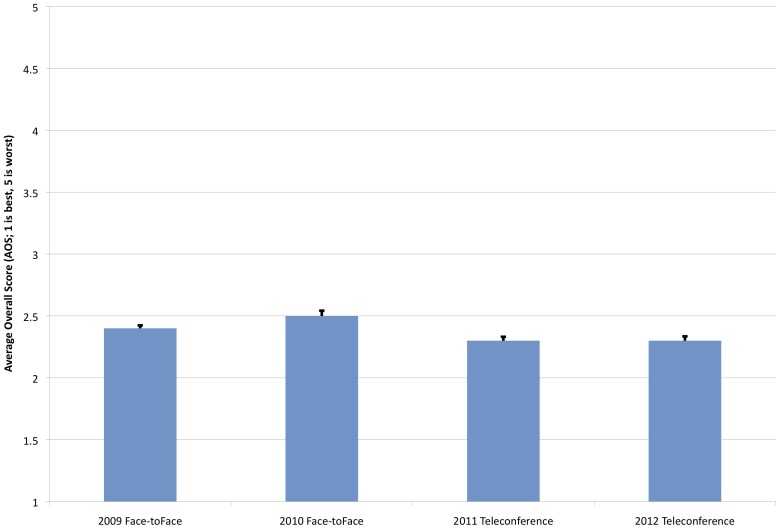
Comparison of Average Overall Score (AOS). Average score comparison between 2009, 2010 (face-to-face) and 2011, 2012 (teleconference) reviews. The total numbers of applications reviewed were 669, 291, 347, and 297 for 2009, 2010, 2011, and 2012, respectively.

Although it is common for standing review panels to develop a corporate memory (particularly with regard to funding level cut-offs) and potentially “chase the pay line” through their scoring, PrX had no standing panels over this timeframe, roughly 50% of reviewers were new from one year to the next [11]. Moreover, PrX had no score cut-off to determine funding because of the wide array of topic areas. Thus, score creep in the AOS for PrX applications over this period is unlikely to play a role in the outcome measures in this analysis. Moreover, as there is no formal resubmission process and the vast majority of applications are first-time submissions, there is a low likelihood of an annual application quality improvement due to resubmission. However, one question we sought to address was whether shifts occurred as a result of the meeting setting (teleconference versus face-to-face). When the AOSs from the 2011 and 2012 teleconference reviews (2.3±0.03 for both years) were compared to those of the 2009 and 2010 face-to-face reviews (2.4±0.02 and 2.5±0.04 respectively), there was only a very slight improvement in scores (**Figure 1**). Some statistically significant difference was found between groups (F[3,1600] = 7.5; p<0.001), specifically between 2010 and 2011 (mean difference = 0.2; p = 0.002, CI: 0.05, 0.33) and between 2010 and 2012 (mean difference = 0.2; p = 0.001, CI: 0.06, 0.35). Neither 2011 nor 2012 data were found to be significantly different from 2009 data (p = 0.12 and 0.07, respectively). The total numbers of applications reviewed were 669, 291, 347, and 297 for 2009, 2010, 2011, and 2012, respectively. It should be noted that a pre-application cull was used in 2010 and 2012 (as mentioned above), which prompted a comparison of AOSs within the same setting to measure the influence of the cull on application quality. The post-hoc Scheffe tests from the ANOVA above yielded no statistical difference between 2009 and 2010 (p = 0.21) or between 2011 and 2012 (p = 0.99). Overall, although there is a rather small improvement in teleconference application scores compared to 2010 face-to-face scores, these data suggest that the teleconference setting does not unduly affect the assessment scoring of applications.

**Figure 2:**
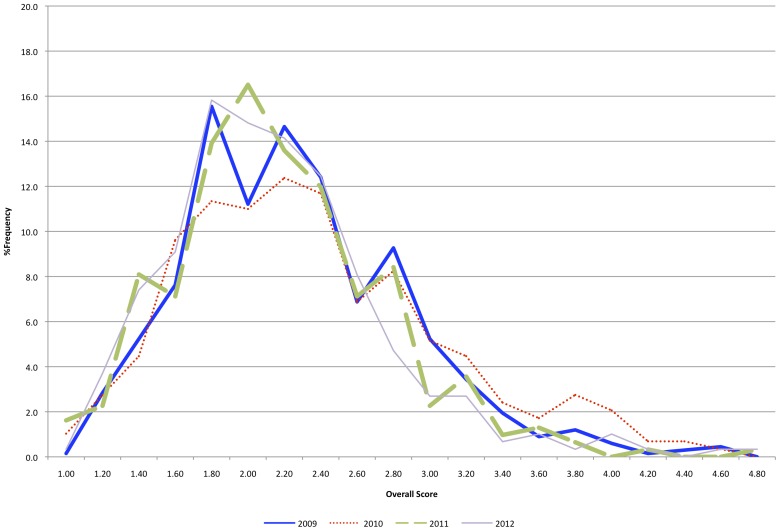
Comparison of Overall Score (OS) Distribution. Overall score (OS) distribution for all applications from 2009, 2010 (face-to-face) and from 2011, 2012 (teleconference) peer reviews.

In addition, the distribution of OSs of all applications in each year was compared (**Figure 2**). Data from 2009 and 2010 (in-person) and from 2011 and 2012 (teleconference) show very little difference. The breadth of the histograms indicates that review panel members made a substantial effort in both face-to-face and teleconference meetings to separate the truly meritorious applications from the poor applications using the full range of the scientific merit scale, providing the funding agency with the information needed to make informed funding choices and giving investigators clear feedback. The score distribution appears to have been largely unaffected by the change to the teleconference review format.

### Average Standard Deviations of Application Scores and Inter-rater Reliability

**Figure 3:**
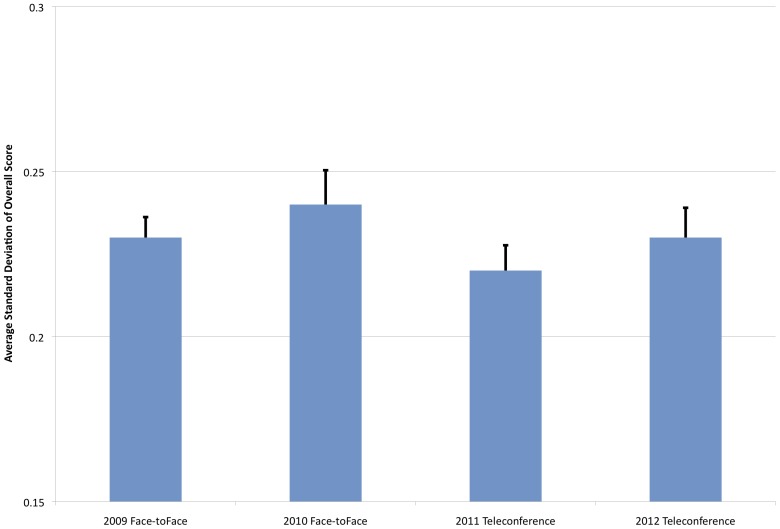
Comparison of Average Standard Deviation of Individual Reviewer Merit Scores. Average standard deviation of individual reviewer merit scores per application, comparing 2009, 2010 (face-to-face) and 2011, 2012 (teleconference) reviews.

The standard deviations of peer review panel member scores for each application have also been recorded. From 1999 to the present, average standard deviations for PrX have remained relatively stable from year to year, ranging from 0.18–0.27, which is consistent with standard deviations observed in NIH peer review panels [12], [13]. If one compares the average standard deviation for reviews in the 2009 and 2010 face-to-face reviews (0.23±0.006 and 0.24±0.010, respectively) and the 2011 and 2012 teleconference reviews (0.22±0.007 and 0.23±0.009, respectively), one finds they are quite stable (**Figure 3**). In fact, analysis by ANOVA reveals no statistical difference between face-to-face and teleconference settings (F[3,1600] = 1.19; p = 0.31). We further examined potential effects of review setting on the extent to which reviewers agreed on their assessments of the applications via IRR. One can approximate the IRR through the use of an intraclass correlation (ICC) statistic described by Cicchetti for the case of peer review of grant applications (R_i_ Model III) based on an average number of reviews per application (in our case 10) [14]. The calculation of the ICC estimates the correlation of reviewers' ratings for a given application (also known as the intra-application correlation or single-rater reliability) [15]. The standard error is calculated using the same methodology as outlined in Ip et al [16].

**Figure 4:**
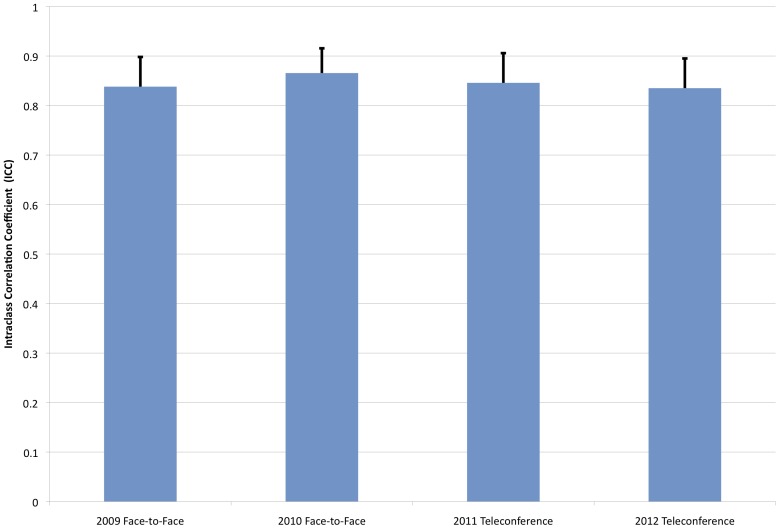
Comparison of Intraclass Correlation. Intraclass correlation for 2009, 2010 (face-to-face) and 2011, 2012 (teleconference) reviews (p<0.01 for all years).

The ICC is plotted for 2009–2010 (face-to-face) and 2011–2012 (teleconference). The ICC is stable, ranging from 0.84 to 0.87 (p<0.01 for all years) with a standard error of approximately 0.06 over all years (**Figure 4**). The reliability of the mean application rating (IRR) is then estimated from the ICC using the Spearman-Brown formula and found to be 0.98 for all years [17]. Both the intra-application correlation and the IRR reported here are higher than some values reported in the literature for panel peer review [14], [18]. This is likely due to our review protocol, in which all reviewers submit an independent score for each application with which they are not in conflict; similar increases have been found by Marsh et al. with similar all-inclusive review protocols versus ad-hoc controls [15]. In addition, the number of reviewers voting per application in our study is much higher than values reported in the literature, and it is known that the reliability of ratings does increase with an increase in the number of raters [19]. In any case, these IRR values indicate that this peer review has a high level of reliability.

There is a small level of variation in the ICC from 2009 to 2012, which is less than the calculated error, and there is no obvious trend observed over time for either the ICC or the IRR. These data suggest that the teleconference review setting does not contribute to the contentiousness of panel decisions and does not drive decisions toward or away from consensus.

### Average Application Discussion Times

**Figure 5:**
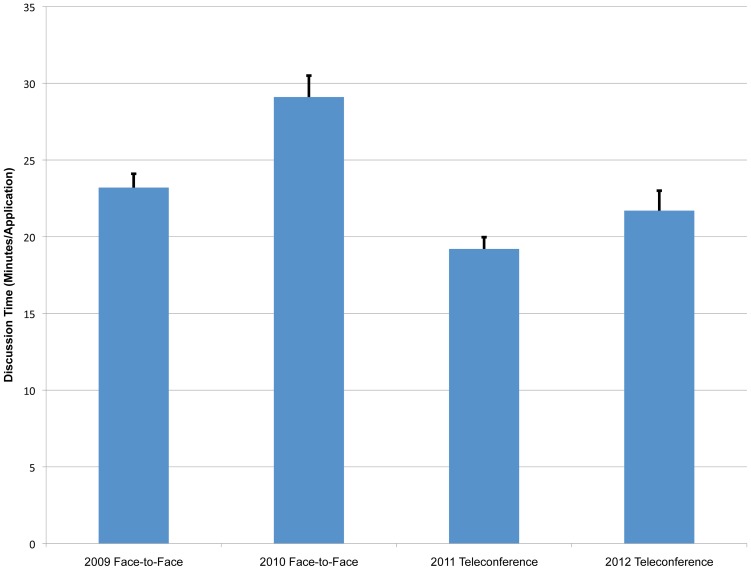
Comparison of Average Discussion Time per Application. Average discussion time per application over all panels for face-to-face (2009–2010) and teleconference (2011–2012) years. In 2009, there was an average of 28 applications per panel, whereas in 2010, 2011 and 2012, there were averages of 18, 16, and 20 applications, respectively, per panel.

The average time spent discussing each application was calculated for each year, and then a comparison was plotted in **Figure 5** between face-to-face (2009–2010) and teleconference (2011–2012). From this graph, it seems averages from 2009 (23±0.9 minutes/application; number of panels = 20), 2011 (19±0.8 minutes/application; number of panels = 19) and 2012 (22±1.3 minutes/application; number of panels = 13) were all fairly comparable, while 2010 (29±1.4 minutes/application; number of panels = 13) averaged longer. In order to explore these differences, we examined the number of applications submitted each year. There is a relationship that we have noted in face-to-face reviews between application number per panel and discussion time, whereby a smaller application load tends to produce longer discussion times; however, this relationship is much more muted (if present at all) for teleconference panels (data not shown). If one compares application numbers, 2009 had an average of 28 applications per panel, whereas 2010, 2011 and 2012 had averages of 18, 15, and 20 applications respectively per panel. Therefore, based on application load, 2010 is more comparable to 2011 and 2012 than is 2009, where the discussion time may have been truncated because of the large number of applications.

However, there does appear to be a statistical difference in average discussion time between face-to-face and teleconference reviews (F[3,61] = 14.54; p<0.001), specifically between 2010 versus 2011(mean difference = 9.97; p<0.001, CI: 5.56, 14.37) and 2010 versus 2012 (mean difference = 7.43; p<0.001, CI: 2.63, 12.24). This difference may be in part due to the review setting, as reviewers are “captive” at face-to-face meetings. This physical restriction may lend itself to extended peripheral discussions. In contrast, discussion during teleconference reviews may be more focused and efficient, as reviewers have a reduced level of interaction and can quickly reengage into their daily activities once the teleconference has ended. It should be noted that no correlation was observed between average panel scores and discussion time (data not shown).

Video teleconferencing has been suggested as a virtual hybrid of face-to-face and teleconference meetings. In order to explore this mechanism for grant application review, AIBS piloted two video teleconference panels in 2011 (with 6 applications reviewed by one panel and 9 applications reviewed by the other). We observed average application discussion times of 15 and 17 minute per application, respectively, which are lower than the overall 2011 and 2012 teleconference averages (19 and 22 minutes/application, respectively), suggesting that video teleconferencing does not avoid the loss in discussion time seen in teleconferences. This observation is consistent with the literature [20].

### Reviewer Recruitment

**Figure 6:**
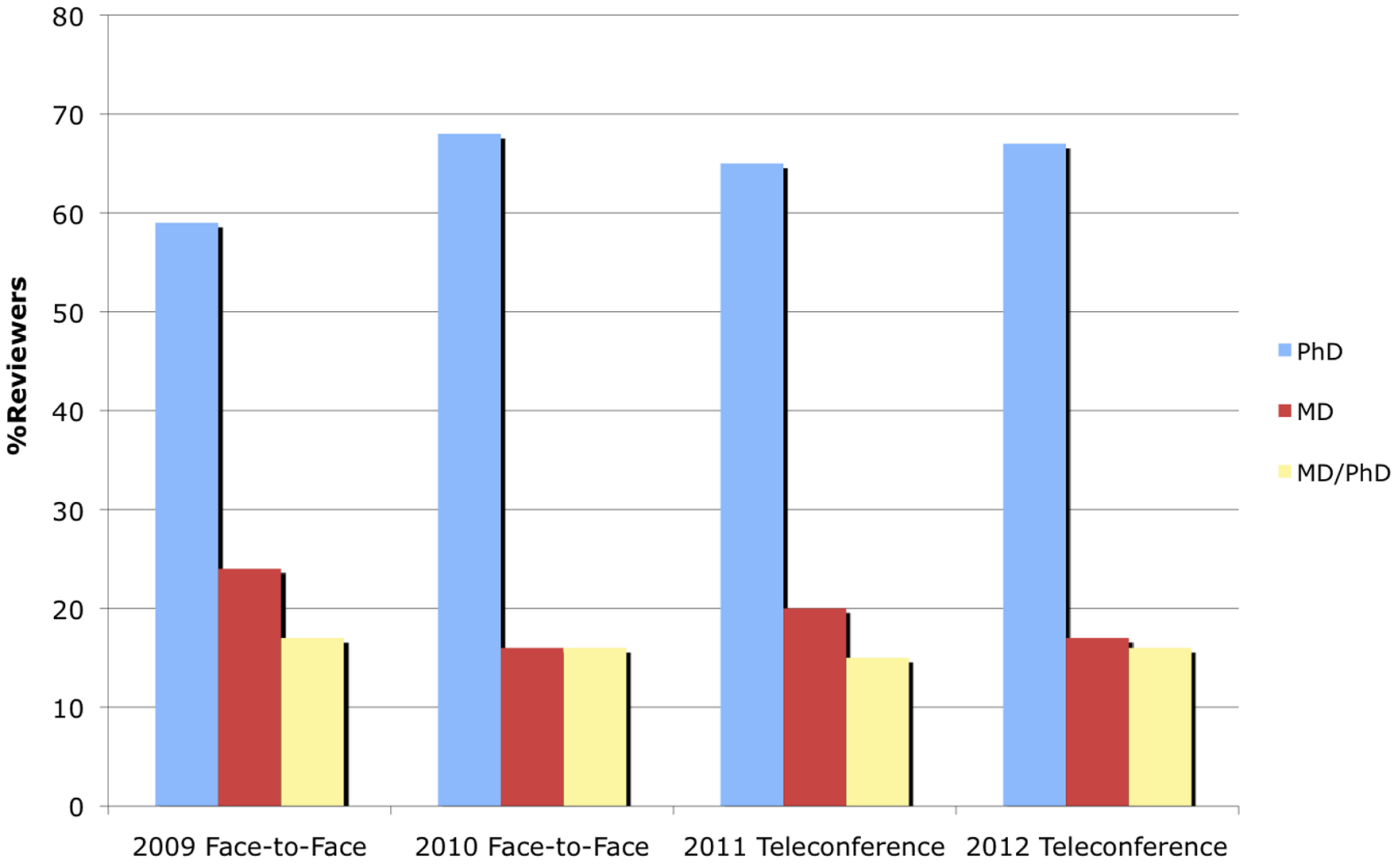
Comparison of Reviewer Degrees. Relative proportions of reviewer degrees for each year (MD, MD/PhD, and PhD).

**Figure 7:**
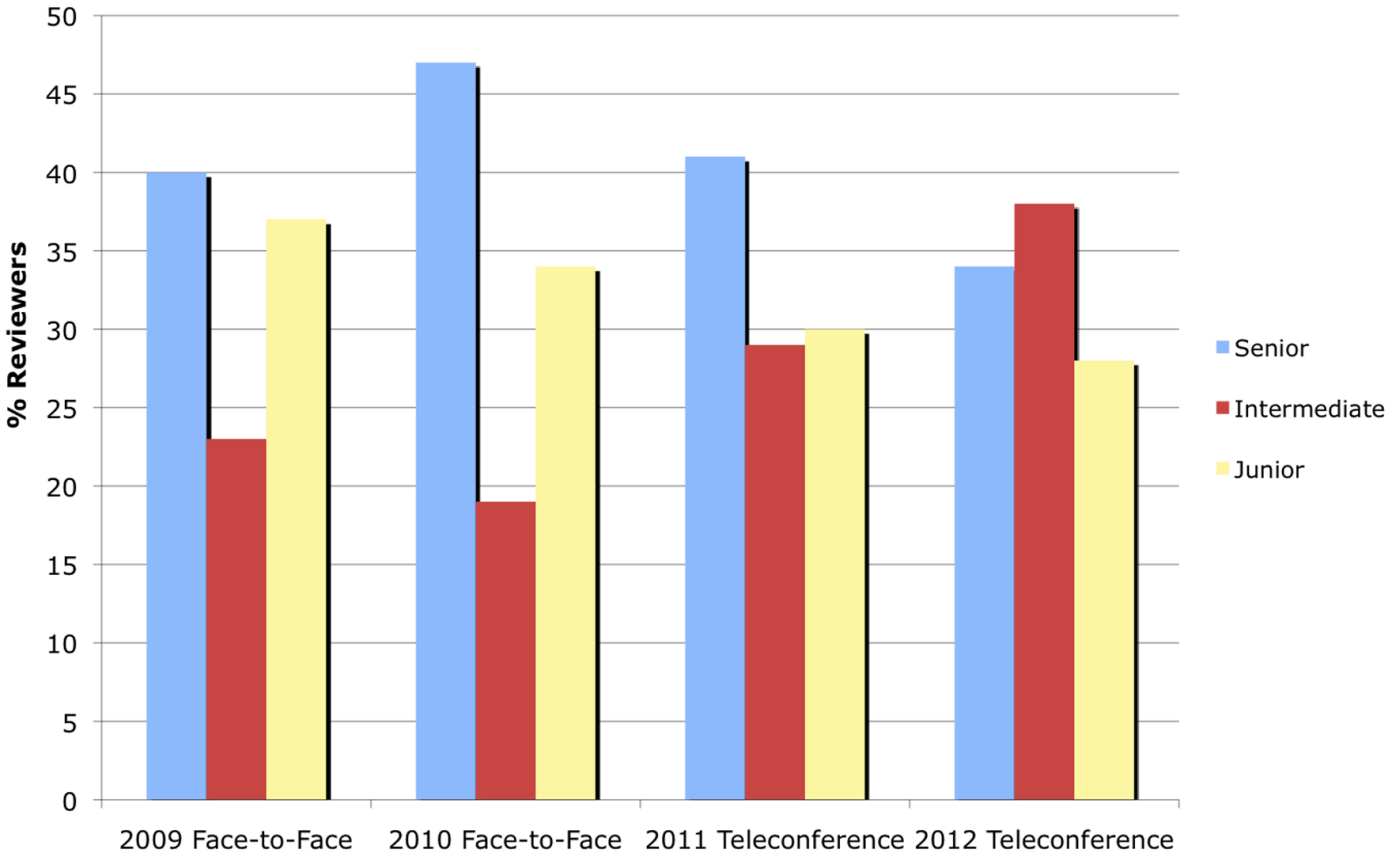
Comparison of Reviewer Seniority. Relative proportion of reviewers in terms of seniority for each year. The senior academic level grouping included full professors, chairs, deans, and/or directors, the intermediate level grouping included associate professors, and the junior level grouping included assistant professors or equivalents.

AIBS staff have also tracked review panel member demographics over time for this program, to get a sense of whether review setting has a significant effect on reviewer recruitment. In **Figure 6** the proportion of review panel members with MD or equivalent only, both MD and PhD, and PhD or equivalent only degrees are plotted over time. These data show that roughly 15–25% held an MD or equivalent degree, 15–20% held an MD/PhD degree, and 55–70% held a PhD or equivalent degree for all years. In terms of seniority of review panel members, the data displayed in **Figure 7** show that roughly 35–45% had achieved a senior academic level or equivalent (Full Professor, Chair, Dean, and/or Director), 30–35% had achieved an intermediate level (Associate Professor) and 20–35% were junior level (Assistant Professor or equivalent) for all years. These distributions seem unaffected by whether the review setting was via teleconference or face-to-face. Given that the type of research proposed was not radically different in scope or clinical relevance over this time span, this suggests that the recruitment of appropriate reviewers (in terms of clinical experience and seniority) was unaffected by review setting.

### Survey Data

AIBS routinely surveys reviewers for quality control purposes and to identify any areas in which improvement can be made. In light of the change in setting from face-to-face to teleconference, we polled reviewers to see if they felt the setting had any influence on the outcome of the review. Survey data from 2012 (N = 90) assessed peer review quality using a questionnaire with answers scored on a scale of 1.0 to 5.0 (where 5 is the best and 1 is the worst).

For the question “To what extent did you find the panel discussions fair and thorough?” an average answer of 4.5 was returned, with 98% of reviewers scoring above a 3.0 (98% of surveyed reviewers answered this question). This question was asked in a 2008 survey of this program (when a face-to-face review setting was employed), which also resulted in an average answer of 4.5.

For the question “Thinking of your past experiences with in-person, on-site review panels, to what extent did the teleconference review panel format achieve a thorough review of each application?” an average score of 4.0 was returned, with 77% of reviewers scoring above a 3.0 (81% of surveyed reviewers answered this question).

These questions relate to the thoroughness and equity of the review discussions themselves. This issue is of importance particularly to the funding agency and to the applicants [21], [22]. Based on the average responses, it is clear that review panel members found panel discussions to be fair and thorough, despite the change in review setting.

In addition to surveying the reviewers about the review process, AIBS also receives feedback from the funding agency. Although the feedback was positive and no change in review quality from the switch from face-to-face to teleconference reviews was noted, AIBS is currently in the process of acquiring more quantitative survey data on this topic.

## Conclusions

The PrX program serves as an interesting and informative case study of the effects of review setting on the metrics of the peer review process. The data indicate that little difference is found in most of the review metrics between face-to-face and teleconference settings, which is consistent with group performance literature [9], [10], [23]. Application scoring was only modestly affected, the contentiousness of opinion was completely unaffected and reviewers used the full scoring range regardless of review setting. The reviewer ratings were found to be highly reliable in both review settings; as noted above, the reliability was found to be higher than what others have reported in the literature, probably due to protocol and the number of assessors per application. In addition, reviewer participation (specifically, the ability to recruit senior or clinician reviewers) was not hampered by review setting. Reviewer feedback has indicated that the AIBS process is viewed as fair and thorough, independent of review setting.

There was some difference noted in terms of discussion time; teleconferencing and, in a much smaller sample, video teleconferencing had shorter discussion times when compared with face-to-face review data. As mentioned above, the captivity of reviewers and the opportunity for interactions at a one- or two-day face-to-face panel meeting likely lends itself to prolonged peripheral discussion, which may not occur in the focused teleconference format. It has been hypothesized that while task-oriented focus is increased in a teleconference setting, there may be lower member engagement in group activities [23]. A structural intervention to improve group decision-making known as the “stepladder technique” (which orders the entry and type of input into a group by participants) has been tested in a teleconference setting and has resulted in improved decision-making performance [23]. This intervention is suggested to work, at least in part, by counteracting decreased participant engagement. There are similarities between the peer review process described here and the stepladder technique, in that reviewer input is structured in a step-wise manner, with a gradual opening up of participant input after a small group has presented.

The importance of potential decreased peripheral discussion and member engagement on the final scoring decisions of teleconference reviews, as well the ultimate productivity of the research funded through this process, is unclear. We have found no correlation of either final application scores or standard deviations with discussion time (data not shown). A few studies have attempted to examine the effects of discussion on reliability and scoring decisions, however the results are mixed and more studies need to be conducted before any clear conclusion can be drawn [12], [18], [24]. Further studies (particularly including the use of prospective trials) should aim to assess any correlation between discussion and member engagement with outcome measures of the peer review process and productivity of funded research projects. Nevertheless, many of the review metrics recorded here display invariance to review setting, which may help in alleviating some fears concerning the quality of teleconference reviews compared to face-to-face reviews. With the substantial benefits that teleconference reviews bring in minimizing travel (reducing environmental impact) and offering substantial cost-savings to the funding agency, more data should be acquired to further validate this review option.
